# Replication and Meta-Analysis of GWAS Identified Susceptibility Loci in Kawasaki Disease Confirm the Importance of B Lymphoid Tyrosine Kinase (*BLK*) in Disease Susceptibility

**DOI:** 10.1371/journal.pone.0072037

**Published:** 2013-08-30

**Authors:** Chia-Jung Chang, Ho-Chang Kuo, Jeng-Sheng Chang, Jong-Keuk Lee, Fuu-Jen Tsai, Chiea Chuen Khor, Li-Ching Chang, Shih-Ping Chen, Tai-Ming Ko, Yi-Min Liu, Ying-Ju Chen, Young Mi Hong, Gi Young Jang, Martin L. Hibberd, Taco Kuijpers, David Burgner, Michael Levin, Jane C. Burns, Sonia Davila, International Kawasaki Disease Genetics Consortium, Korean Kawasaki Disease Genetics Consortium, Taiwan Kawasaki Disease Genetics Consortium, Yuan-Tsong Chen, Chien-Hsiun Chen, Jer-Yuarn Wu, Yi-Ching Lee

**Affiliations:** 1 Graduate Institute of Microbiology, College of Medicine, National Taiwan University, Taipei, Taiwan; 2 Institute of Biomedical Sciences, Academia Sinica, Taipei, Taiwan; 3 Department of Pediatrics, Kaohsiung Chang Gung Memorial Hospital, Kaohsiung, Taiwan; 4 Graduate Institute of Clinical Medical Science, Chang Gung University College of Medicine, Kaohsiung, Taiwan; 5 Department of Pediatrics, China Medical University and Hospital, Taichung, Taiwan; 6 Asan Institute for Life Sciences, University of Ulsan College of Medicine, Seoul, Korea; 7 School of Chinese Medicine, China Medical University, Taichung, Taiwan; 8 Department of Medical Genetics, China Medical University Hospital, Taichung, Taiwan; 9 Department of Health and Nutrition Biotechnology, Asia University, Taichung, Taiwan; 10 Division of Human Genetics, Genome Institute of Singapore, Singapore; 11 Department of Ophthalmology, School of Medicine, National University of Singapore, Singapore; 12 Department of Paediatrics, School of Medicine, National University of Singapore, Singapore; 13 Department of Pediatrics, Ewha Womans University Hospital, Seoul, Korea; 14 Department of Pediatrics, Korea University Hospital, Ansan, Korea; 15 Division of Infectious Diseases, Genome Institute of Singapore; 16 Department of Pediatric Hematology, Immunology and Infectious Diseases, Emma Children’s Hospital Academic Medical Center, Amsterdam, The Netherlands; 17 Murdoch Childrens Research Institute, The Royal Children’s Hospital, Parkville, Victoria, Australia; 18 Department of Paediatrics, University of Melbourne, Victoria, Australia; 19 Department of Pediatrics, Imperial College London, London, United Kingdom; 20 Department of Pediatrics, University of California San Diego School of Medicine, La Jolla, California, United States of America; 21 School of Epidemiology and Public Health, National University of Singapore, Singapore ,¶ A complete list of members and affiliations appears in File S1; 22 Department of Pediatrics, Duke University Medical Center, Durham, North Carolina, United States of America; 23 Institute of Molecular Medicine, National Tsing Hua University, Hsinchu, Taiwan; 24 Institute of Cellular and Organismic Biology, Academia Sinica, Taipei, Taiwan; National Taiwan University Hospital, Taiwan

## Abstract

The *BLK* and *CD40* loci have been associated with Kawasaki disease (KD) in two genome-wide association studies (GWAS) conducted in a Taiwanese population of Han Chinese ancestry (Taiwanese) and in Japanese cohorts. Here we build on these findings with replication studies of the *BLK* and *CD40* loci in populations of Korean and European descent. The *BLK* region was significantly associated with KD susceptibility in both populations. Within the *BLK* gene the rs2736340-located linkage disequilibrium (LD ) comprising the promoter and first intron was strongly associated with KD, with the combined results of Asian studies including Taiwanese, Japanese, and Korean populations (2,539 KD patients and 7,021 controls) providing very compelling evidence of association (rs2736340, OR = 1.498, 1.354–1.657; *P* = 4.74×10^−31^). We determined the percentage of B cells present in the peripheral blood mononuclear cell (PBMC) population and the expression of *BLK* in the peripheral blood leukocytes (leukocytes) of KD patients during the acute and convalescent stages. The percentage of B cells in the PBMC population and the expression of *BLK* in leukocytes were induced in patients in the acute stage of KD. In B cell lines derived from KD patients, and in purified B cells from KD patients obtained during the acute stage, those with the risk allele of rs2736340 expressed significantly lower levels of BLK. These results suggest that peripheral B cells play a pathogenic role during the acute stage of KD. Decreased BLK expression in peripheral blood B cells may alter B cell function and predispose individuals to KD. These associative data suggest a role for B cells during acute KD. Understanding the functional implications may facilitate the development of B cell-mediated therapy for KD.

## Introduction

Kawasaki disease (KD) (OMIM 300530) is an acute, self-limited vasculitis predominantly affecting infants and young children [1]. The disease is characterised by prolonged fever and at least four out of five diagnostic features: polymorphous skin rash; bilateral conjunctival injection; erythema of the oral mucosa, lips, and tongue; erythema and red skin on the palms of the hands and the soles of the feet; and cervical lymphadenopathy. Coronary artery aneurysms develop in 15–25% of untreated patients, making KD the leading cause of acquired heart disease in children in developed countries [2]. Treatment with intravenous immunoglobulin (IVIG) abrogates the inflammation in approximately 80% of affected individuals and reduces the incidence of coronary artery lesions to <5%. Coronary artery aneurysms may lead to ischaemic heart disease, myocardial infarction, and sudden death. Clinical and epidemiological findings suggest that an infectious agent triggers an inflammatory response that leads to host immune dysregulation in genetically predisposed individuals; however, no pathogen has been isolated, and the aetiology of KD remains unknown.

Multiple lines of evidence suggest that genetic determinants contribute to KD susceptibility and outcome. Asian countries have a much higher incidence of KD than Western countries and Taiwan has the third-highest annual incidence rate after Japan and Korea. Genome-wide association studies (GWAS) conducted in Japanese [3], Han Chinese descendants in Taiwan (Taiwanese) [4], Korean [5], and European [6] populations have identified several biologically plausible candidates for KD susceptibility and coronary artery lesions. SNPs in the *BLK* and *CD40* loci have been implicated in KD in two recent GWAS conducted in Taiwanese and Japanese populations [3], [4]. To confirm the association of *BLK* and *CD40* with KD, we conducted replication studies in populations of Korean- and European-descent and performed a meta-analysis of the current and previously published studies. The linkage disequilibrium (LD) block, comprising the promoter region of *BLK* showed the most significant association with KD in the combined Asian studies. Additionally, we examined the percentage of B cells in the peripheral blood mononuclear cell (PBMC) population and the levels of BLK expression in peripheral blood leukocytes (leukocytes) from KD patients at different stages of disease development. We further examined the allelic regulation of KD-associated SNPs in *BLK* expression and the possible regulation of downstream signaling of B cell receptor stimulation. Our results suggest a possible role involvement of B cells in immune homeostasis and KD pathogenesis.

## Materials and Methods

### Patients and Samples

Kawasaki disease (KD) patients and controls from populations of Taiwanese, Korean, Japanese, and European patients have been described in detail previously [3], [4], [5], [6]. All KD subjects were diagnosed according to accepted criteria for KD [7], [8]. Blood samples of different stages of KD were enrolled by Kaohsiung Chang Gung Memorial Hospital, Kaohsiung, Taiwan, and China Medical University Hospital, Taichung, Taiwan. The samples were collected from patients in the acute stage (within 24 h before IVIG treatment, KD1), after IVIG treatment (3–7 days after IVIG treatment, KD2), and during the convalescence stage (3 weeks after IVIG treatment, KD3). All patients were initially treated with a single dose of IVIG (2 g/kg) over a 12 hour period. Lymphoblastoid cell lines from KD patients were established by transforming peripheral B lymphocytes with Epstein-Barr virus. The age-matched febrile controls (FC) were enrolled by Kaohsiung Chang Gung Memorial Hospital, Kaohsiung, Taiwan. They were admitted for upper and/or lower respiratory tract infections (including acute bronchiolitis, acute pharyngitis, acute bronchitis, croup, and acute tonsillitis). The studies were approved by the institutional review boards and ethics committees of all institutions. Written informed consent was obtained from the subjects’ parents in accordance with institutional requirements and Declaration of Helsinki principles.

### Genotyping

The genotypes of the Taiwanese and Korean collections for the GWAS cohorts of the study were analysed with the Affymetrix Genome-Wide Human SNP Array 6.0 [4], [5] and those of the Japanese and European populations with Illumina Human Hap550v3 BeadChip and Illumina Human 610K Quad BeadChips, respectively [3], [6]. For the replication study of the *BLK* SNP rs2736340 in the Japanese populations, genotypes were detected by direct sequencing [3]. The genotype data of rs2736340 in Taiwanese and Japanese patients were publically available [3], [4]. The three tag SNPs (rs2736340, rs6993775, and rs1382566) in *BLK* selected for the replication phase in the Korean collection were genotyped with the ABI TaqMan allelic discrimination assay.

### Statistical Analysis

Genotype data of the tested SNPs in the cases and controls were directly obtained from participating studies (Taiwanese, Korean, and International Kawasaki studies). Cochran–Armitage trend *P* values and allele frequencies were then generated based on the genotype frequencies. The data from the Japanese study was obtained from the authors’ previously published data [3]. A meta-analysis was then performed using a weighted average method with inverse-variance weights: w = 1/se2. An overall *z*-statistic and *P* value was then calculated from the weighted average of the individual statistics. The meta-analysis was performed with METAL (http://www.sph.umich.edu/csg/abecasis/Metal). Meta-analyses of the tested SNPs were carried out in three phases based on the combined data of (1) Taiwanese and Japanese Kawasaki GWAS; (2) all Asian studies, including Taiwanese, Japanese, and Korean studies; and (3) all Asian studies and the international Kawasaki study. Cross-study heterogeneity assuming fixed effects were examined with the heterogeneity index I^2^, implemented in PLINK 1.07.

### PBMC Isolation, Lymphocyte Subsets, and B Cell Preparation

The peripheral blood mononuclear cells (PBMCs) were isolated from heparinised blood from KD patients at different stages (as described in the Patients and Samples section) by density gradient sedimentation using Ficoll-Hypaque (Histopaquen-1077, Sigma-Aldrich, St. Louis, MO). The percentages of B cell (CD19+) and T cell (CD3+) subsets were determined by multicolor flow cytometry with a FACSCalibur (BD Biosciences) using monoclonal antibodies against CD3 (UCHT1; BD Biosciences, Mississauga, Ontario, Canada) and CD19 (clone HIB19; eBioscience). Data were analyzed with CellQuest acquisition software (BD Biosciences). B cells were isolated using anti-CD19-coated magnetic beads (Dynabeads M450 Pan B; Life Technologies, NY, USA).

### Real-time PCR

Total RNA from the peripheral blood leukocytes (leukocytes) of KD patients at different stages of disease development or from age-matched fever controls were isolated with the FavorPrep Blood/Cultured Cell Total RNA Purification Kit (Favorgen). Total RNA samples from lymphoblastoid cell lines (peripheral B lymphocytes transformed by Epstein-Barr virus) of different allele types of rs2736340 established from KD patients were isolated using TRIzol reagent (Life Technologies). Reverse transcription was performed with the SuperScriptIII First-Strand Synthesis System (Life Technologies). BLK mRNA expression levels were detected by real-time RT-PCR using SYBR Green PCR Master Mix and the ABI Prism 7900 HT Sequence Detection System (Applied Biosystems). BLK expression levels were normalized to 18 S in the PBLs study and to GAPDH in lymphoblastoid cell lines. The final results were presented as relative expression levels. The primers used for amplifying BLK mRNA were 5′-CTT CAC CAT CAA AGC AGA CG-3′ (forward) and 5′-CTC CAG GTT GCG GAT GAC-3′ (reverse). The primers used for amplifying 18S mRNA were 5′-GTA ACC CGT TGA ACC CCA TT-3′ (forward) and 5′-CCA TCC AAT CGG TAG TAG CG-3′ (reverse). The primers used for amplifying GADPH mRNA were 5′-TTC GCT CTC TGC TCC TCC TGT-3′ (forward) and 5′-GCC CAA TAC GAC CAA ATC CG-3′ (reverse).

### Western Blot

Total protein lysates were isolated from lymphoblastoid cell lines established from KD patients and purified B cells from acute stage KD patients with different allele types of rs2736340. Proteins (4–20 µg per lane) were separated by standard SDS-PAGE and then transferred onto PVDF membranes and probed with the following antibodies: BLK (sc-329, Santa Cruz Biotechnology), ERK1/2 C-16 (SC-123; Santa Cruz Biotechnology), phospho-p44/p42 MAPK (ERK1/2) Thr220/Thr204 (9101; Cell Signaling), GAPDH (14C10; Cell Signaling). Horseradish peroxidase–conjugated secondary antibodies were then used, followed by detection with a chemiluminescence detection system (Amersham Biosciences).

### Expression Quantitative Trait Locus (eQTL) Analysis

The correlation between HapMap genotypes (HapMap3 release#3, coded according to NCBI build 36 on the forward strand, 1.46 million SNPs) and gene expression levels (GENEVAR project, using genome-wide expression arrays including 47294 transcripts normalized independently for each population or all together [9]) in EBV-transformed B-cell lines from the same 270 HapMap individuals was generated using the web-based tool SNPexp v1.2 [10] (http://app3.titan.uio.no/biotools/tool.php?app=snpexp). The 270 HapMap individuals from 4 populations include: 45 unrelated Han Chinese in Beijing (CHB), 45 unrelated Japanese in Tokyo (JPT), 90 (30 trios) individuals of Utah residents with ancestry from northern and western Europe (CEU), and 90 (30 trios) Yoruba individuals of Ibadan, Nigeria (YRI). The correlations were analyzed with the additive genotypic model without adjustment for multiple testing.

## Results

### Replication Studies in Korean and European Populations Validate Previous Genetic Associations in KD and Implicate an LD Region within the *BLK* Promoter

**Figure 1:**
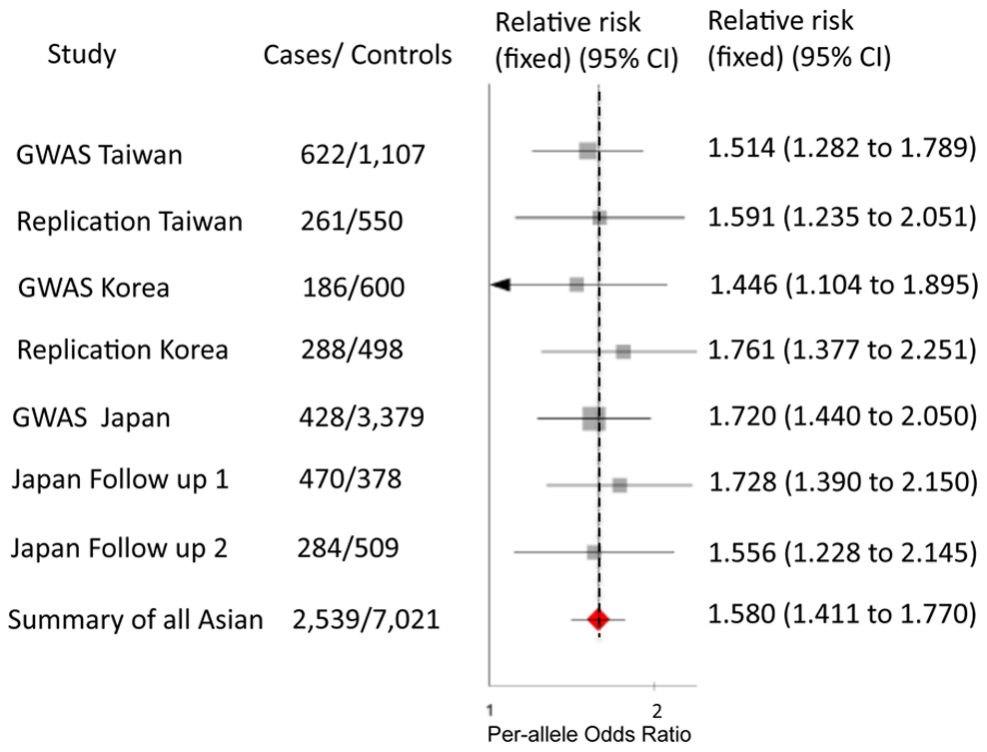
Per-cohort analysis of *BLK* rs2736340. Boxes indicate the odds ratio for each cohort, and horizontal lines denote the 95% confidence interval for the corresponding odds ratio. Diamonds represent summary odds ratios for the respective meta-analyses. Solid vertical line indicates an odds ratio of 1.0, and dashed vertical line denotes the odds ratio obtained from meta-analysis of all samples.

Both *BLK* and *CD40* loci have been identified as having the most significant associations with KD in two GWAS conducted in Taiwanese and Japanese populations [3], [4]. To further validate the associations, we performed replication studies of these two loci in a Korean cohort comprising 186 patients with KD and 600 healthy controls, previously genotyped by Affymetrix SNP Array 6.0 for GWAS [5]. Twelve SNPs in *BLK* were associated with KD in this Korean cohort (*P*<0.05) (Table S1 in File S1). These significantly associated SNPs in the *BLK* gene were distributed in neighbouring LD blocks, including the promoter and intron 1 of *BLK* (Figure S1 in File S1). Based on LD structure (Figure S1 in File S1), we evaluated three tag SNPs, rs2736340, rs6993775, and rs1382566, in a separate independent Korean cohort, composed of 288 children with KD and 498 controls. These three SNPs all showed significant associations with KD in the second Korean replication cohort (*P*<0.0001; Table 1). The SNP rs2736340 located in the LD region of the *BLK* promoter showed the most significant association with KD in Korean populations. Additionally, a meta-analysis of four independent sets from Taiwanese and Korean populations confirmed the association (*P* = 1.41×10^−15^; Table 1). We then performed a meta-analysis using rs2736340 data from the current replication studies in Korean populations with previously published data from Taiwanese and Japanese populations. This Asian meta-analysis gave compelling evidence of association with KD (rs2736340, OR = 1.498, 95% CI, 1.354–1.657; *P* = 4.74 × 10^−31^; Table 2 and Figure 1). The cross-study heterogeneity was examined by the I^2^ index (see Methods). The index was *I^2^* = 0.0, suggesting low heterogeneity among these tested Asian groups.

**Table 1:** Association between genetic variants in the *BLK* region and KD in a combined analysis of Taiwanese and Korean populations.

						RAF			
SNPs	Chr	Position^a^	Locus	Risk Allele		Control	Case	OR^g^ (95%CI)	*P* value
rs2736340	8	11381382	*BLK*	T	GWAS Taiwan^b^	0.722	0.797	1.514 (1.282–1.789)	8.74×10^−7^
				T	Replication Taiwan^c^	0.718	0.802	1.591 (1.235–2.051)	2.56×10^−4^
				T	GWAS Korea^d^	0.694	0.766	1.446 (1.104–1.895)	5.23×10^−3^
				T	Replication Korea^e^	0.696	0.801	1.761 (1.377–2.251)	4.03×10^−6^
				T	Meta-*P* f				1.41×10^−15^
rs6993775	8	11407398	*BLK*	A	GWAS Taiwan	0.719	0.783	1.409 (1.197–1.660)	3.86×10^−5^
				T	Replication Taiwan	0.726	0.797	1.486 (1.155–1.912)	1.74×10^−3^
				A	GWAS Korea	0.706	0.785	1.521 (1.153–2.006)	2.62×10^−3^
				T	Replication Korea	0.714	0.791	1.516 (1.187–1.936)	6.91×10^−4^
				A	Meta-*P*				9.21×10^−12^
rs1382566	8	11422250	*BLK*	G	GWAS Taiwan	0.738	0.800	1.418 (1.198–1.679)	4.40×10^−5^
				C	Replication Taiwan	0.736	0.799	1.425 (1.107–1.835)	5.62×10^−3^
				G	GWAS Korea	0.731	0.819	1.663 (1.240–2.232)	6.08×10^−4^
				G	Replication Korea	0.736	0.809	1.520 (1.183–1.954)	8.25×10^−4^
				G	Meta-*P*				1.40×10^−5^

a SNP positions are derived from NCBI human genome reference sequence Build 36.3.

b 622 KD cases and 1,107 controls.

c 261 KD cases and 550 controls.

d 186 KD cases and 600 controls.

e 288 KD cases and 498 controls.

f Meta-analyses with the Cochran*–*Mantel–Haenszel method.

g These statistical values are for the allelic model. Chr, chromosome; Case RAF, risk allele frequency in KD cases; Control RAF, risk allele frequency in controls; OR, odds ratio; 95%CI, 95% confidence interval.

**Table 2:** Meta-analysis of association of rs2736340 with KD.

Marker (Allele)	Collection	Cases	Controls	CaseRAF	Control RAF	OR (95% CI)	*P* a	Meta-OR (95% CI)	Meta-*P* b
rs2736340 (C/T risk allele: T)	GWAS Taiwan	622	1,107	0.797	0.722	1.514 (1.282–1.789)	8.74×10^−7^		
	Replication Taiwan	261	550	0.802	0.718	1.591 (1.235–2.05)	2.56×10^−4^		
	GWAS Japan	428	3,379	0.778	0.693	1.72 1.44–2.05)	1.3×10^−9^		
	Japan follow-up 1	470	378	0.786	0.68	1.73 (1.39–2.15)	1.3×10^−6^		
	Japan follow-up 2	284	509	0.77	0.693	1.56 (1.23–2.15)	3.1×10^−4^		
	Summary of Taiwanand Japan	2,65	5,923						3.97×10^−25^
	GWAS Korea	186	600	0.766	0.94	1.446 (1.104–1.895)	5.23×10^−3^		
	Replication Korea	288	498	0.801	0.696	1.761 (1.377–2.251)	4.03×10^−6^		
	Summary of all Asian	2,539	7,021					1.580 (1.411–1.770)	4.741×10^−31^
	European descent^c^	05	6252	0.290*	0.251	1.192 (1.011–1.405)	3.66×10^−2^		

a These statistical values are for the allelic model.

b Meta-analyses with the Cochran–Mantel–Haenszel method. Case RAF, risk allele frequency in KD cases; Control RAF, risk allele frequency in controls; OR, odds ratio; 95% CI, 95% confidence interval.

c T is the risk allele, and also the minor allele in European descent.

We further examined the association of *BLK* and *CD40* loci with KD in a cohort of European descent comprising 405 KD patients and 6,252 controls who were previously genotyped by Illumina Human610K Quad BeadChips for GWAS [6]. Significant heterogeneity among Asian- and European-descended populations was observed (*I^2^* = 32.95). Three SNPs (rs12680762, rs2736340, and rs2628476) located in the *BLK* promoter region showed significant association with KD with a *P* value of <0.05 (Table S2 in File S1 and Table 2), whereas the two tagging SNPs (rs6993775 and rs1382566) located in the first intron did not show any similar association.

### The B Cell Population is Highly Induced in Peripheral Blood Mononuclear Cells (PBMC) at the Acute Stage of KD

**Figure 2:**
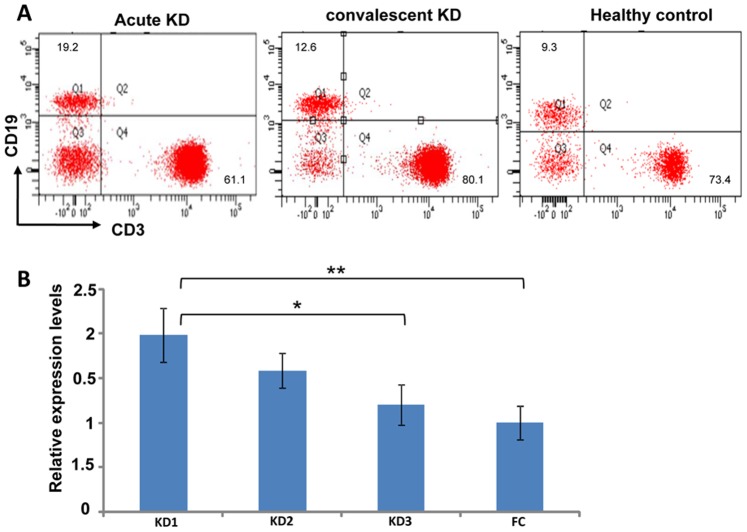
B cell population in peripheral blood mononuclear cells (PBMC) and *BLK* expression in peripheral blood leukocytes (PBLs) induced at the acute stage of KD. (A) PBMCs were stained with anti-CD19 or anti-CD3 monoclonal antibodies, and the percentage of CD19^+^ B cells and CD3^+^ T cells in samples taken from a patient at acute and convalescent stages of KD and from a healthy control were determined by multicolor flow cytometry. (B) Levels of *BLK* expression were determined by real-time RT-PCR, and levels in KD patients were compared to those in fever controls. Values are expressed as mean ± standard error (SE). RNA was harvested from PBLs from KD patients at different stages of disease development or from age-matched fever controls. KD1, *n* = 20, before IVIG treatment (within 24 h before IVIG treatment); KD2, *n* = 12, after IVIG treatment (3–7 days after IVIG treatment); and KD3, *n* = 10, convalescence stage (3 weeks after IVIG treatment). FC, *n* = 19, fever controls.

*BLK*, a src family tyrosine kinase expressed primarily in the B cell lineage [11], transduces signals downstream following stimulation of B cell receptors. It is important for establishing the B cell repertoire during development of B cells [12], and it might also have a role in B cell activation in peripheral blood. To determine how B cells might correlate and functionally contribute to KD development, we first determined the percentage of CD19^+^ B cells and CD3^+^ T cells in the PBMC population at acute and convalescent stages of KD. The percentage of B cells was significantly increased during the acute stage and decreased at the convalescent stage of KD. In contrast, the percentage of T cells was decreased in the PBMCs at the acute stage of KD (Figure 2A). The BLK expression pattern at acute and convalescent stages in peripheral blood leukocytes (leukocytes) correlated with the percentage of B cells in the PBMCs (Figure 2B). To minimize the amount of blood required in further studies, we determined the *BLK* expression in the leukocytes. The expression of *BLK* in the leukocytes was significantly induced at the acute stage in KD patients compared to age-matched fever controls, and the expression levels were decreased after IVIG treatment and further reduced at the convalescent stage (Figure 2B). These results strongly indicate the possible involvement of B cells in immune homeostasis and the development of KD.

### rs2736340 is Associated with BLK Expression in B Cells

To determine whether KD–associated SNPs in the *BLK* region affects the transcript abundance of *BLK*, we examined whether expression of *BLK* correlated with the genotypes of three tag SNPs within the *BLK* gene in B-cell lines from 45 Han Chinese in Beijing (CHB) individuals using a publicly available resource (web-based tool SNPexp v1.2) that used an expression quantitative trait locus (eQTL) generated from transformed B-cell lines by HapMap (see Methods). The results showed that of the three tag SNPs, rs2736340 was the most significantly associated with *cis* expression of the *BLK* gene (*P* = 1.379×10^−4^; Table S3 in File S1). We then analyzed expression of *BLK* correlated in relation to rs2736340 genotypes in B-cell lines from Japanese in Tokyo (JPT), samples from Utah residents with ancestry from northern and western Europe (CEU), and samples taken from Yoruba subjects in Ibadan, Nigeria (YRI). These analyses showed that the individuals carrying the risk allele T of rs2736340 exhibited significantly lower expression of *BLK* in most of the populations tested except for the results from YRI parents (Table 3).

**Table 3:** Analysis of the correlation of genotypes of rs2736340 in *BLK* region with expression levels in transformed B cells.

SNP rs2736340	Geno	C/C	C/T	T/T	P value
CHB	Counts	3	17	23	1.379×10^−4^
	Freq	0.067	0.395	0.535	
	Mean	10.630	10.350	9.647	
	SD	0.583	0.470	0.644	
JPT	Counts	5	25	14	2.665×10^−3^
	Freq	0.119	0.548	0.333	
	Mean	10.750	10.390	9.847	
	SD	0.520	0.570	0.760	
CEU_parents	Counts	28	26	2	1.521×10^−4^
	Freq	0.500	0.464	0.004	
	Mean	11.170	10.580	10.270	
	SD	0.463	0.677	0.059	
CEU_children	Counts	16	6	3	5.005×10^−6^
	Freq	0.640	0.240	0.120	
	Mean	11.070	10.280	9.465	
	SD	0.483	0.509	0.406	
YRI_parents	Counts	46	9	2	7.387×10^−1^
	Freq	0.807	0.158	0.035	
	Mean	10.770	10.760	10.570	
	SD	0.599	0.601	0.154	
YRI_children	Counts	19	8	0	6.654×10^−4^
	Freq	0.707	0.296	0	
	Mean	10.910	10.060	NA	
	SD	0.438	0.689	NA	

Geno, genotypes; Freq, frequency; SD, standard deviation; CHB, 45 Han Chinese in Beijing; JEP, 44 Japanese in Tokyo; CEU_parents; CEU_children; YRI_parents; YRI_children; All populations, 270 individuals from 4 populations (CEU: 90 (30 trios) Utah residents with ancestry from northern and western Europe; CHB: 45 unrelated Han Chinese in Beijing; JPT: 45 unrelated Japanese in Tokyo; YRI: 90 (30 trios) Yoruba in Ibadan, Nigeria). All unrelated, 210 unrelated individuals, including 60 Yoruba (YRI) and 60 CEPH (CEU) parents, and 90 unrelated Chinese (CHB) and Japanese (JPT) samples.

**Figure 3:**
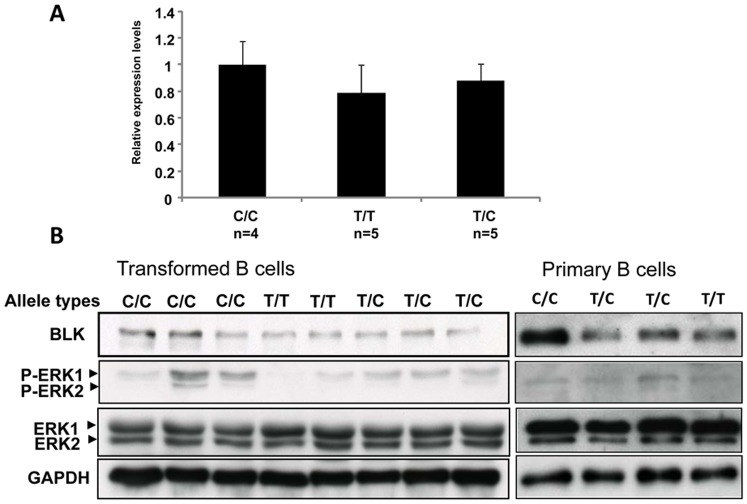
Genotypes of rs2736340 are associated with BLK expression and down stream signaling of B cell receptor in B cell lines established from KD patients and in B cells purified from the acute stage of KD patients. (A) BLK expression was detected by real-time RT-PCR, and expression from patients with T/T or T/C genotypes was compared to that from patients with C/C genotypes. Values are mean ± SE (C/C, *n* = 4; T/T, *n* = 5; and T/C, *n* = 5). (B) BLK expression and ERK1/2 expression and activation were detected by Western blot. BLK, antibody against BLK; ERK1/2, antibody against p44/p42 ERK1/2; p- ERK1/2, antibody against phospho-p44/p42 ERK1/2; GAPDH, antibody against GAPDH.

To validate the correlation between rs2736340 genotypes and *BLK* expression in B cells, we examined the *BLK* expression in B cell lines established from KD patients and in B cells purified from the acute stage of KD patients. We observed that those carrying the risk allele T of rs2736340 express lower levels of BLK (Figure 3A and B). To determine whether BLK expression was associated with B cell receptor downstream signaling, we examined the phosphorylation status of ERK (extracellular signal-regulated kinase). The activation of ERK has been shown to be a signal integration point of downstream B cell receptor stimulation [13]. Reduced ERK activation was observed in B cell lines carrying the rs2736340 risk allele and with low level *BLK* expression (Figure 3B, left panel). However there was no association between ERK phosphorylation status and risk allele of possession of the rs2736340 risk allele in primary B cells purified from the acute stage of KD patients (Figure 3B, right panel).

## Discussion

In this study, we have validated the association between polymorphism of the *BLK* locus with susceptibility to KD in Korean- and European-descended populations. We have narrowed down the rs2736340-located LD block comprising the promoter region of *BLK* that is most significantly associated with KD in a meta-analysis of Asian data from Taiwanese, Japanese, and Korean populations. Fine-mapping and sequencing studies, as well as functional studies are required to identify and validate the potential causal variants.

We observed that *BLK* expression was significantly induced in leukocytes at the acute stage of KD in patients and that expression was reduced during the convalescence stage, which was correlated with the change of B cell population in PBMCs. We also investigated the possible regulatory role of rs2736340. A significant association was found between rs2736340 and *cis* expression of the *BLK* gene in transformed B-cell lines from HapMap data, in transformed B-cell lines derived from KD patients, and in purified B cells from patients was observed. Furthermore, we demonstrated that the risk variant of rs2736340 in *BLK* was associated with regulation of the expression of *BLK* and activation of B cell receptor stimulation in the transformed B-cell lines established from KD patients. However, due to the limited blood volumes that could be obtained during the acute stage in pediatric KD patients, the sample size of studies performed in primary B cells was small and we could not discern a clear trend of the association between risk variant of rs2736340 with the regulation of B cell receptor stimulation. Previously, the risk allele in *BLK* related to systemic lupus erythematosis has been shown to be associated with reduced expression of the *BLK* mRNA transcript in transformed human B cell lines [14]. It has been proposed that the predisposition to autoimmunity associated with low *BLK* expression may reflect involvement of the B cell repertoire established during B cell development. Studies of the cell type-specific *cis* eQTL in purified primary monocytes and B cells from 288 healthy volunteers demonstrated that the *cis*-eQTL of *BLK* was B cell specific [15], suggesting that the allelic regulation of *BLK* expression in the peripheral blood B cells may have physiological roles in immune homeostasis. In this study, we provide evidence linking the risk allele in *BLK* to the reduced expression of *BLK* mRNA transcript and protein, which might correlate with the reduced down-stream signaling of B cell receptor stimulation in peripheral blood B cells. These results suggest that lower expression of *BLK* in peripheral blood B cells during the acute stage is associated with increased risk of KD.

B cells are essential for humoral immunity. In addition to their role in positively regulating immune responses by producing antigen-specific antibody and inducing optimal CD4^+^ T-cell activation, recent studies have revealed that B cells have the ability to negatively regulate cellular immune responses and inflammation [16]. B cells repress T cell proliferation and the differentiation of pro-inflammatory Th1 cells through the CD40-CD40 L dependent pathway [17]. Regulatory B cell control of autoimmunity [18] and inflammation [19] in mice has recently been described, and reduced or absent B cell function exacerbates disease symptoms in autoimmune diseases and acute inflammation [16]. In response to infectious stimuli, a pool of antigen-specific B cells and regulatory B cells quickly proliferate and may inhibit acute inflammation. The expression of lower levels of BLK in B cells impairs the signaling of B cell receptor stimulation and might affect the immune homeostasis and inflammation through two different mechanisms. First, the deficiency of B cell receptor stimulation can affect the antibody secretion and impair host defense. Secondly, the decrease in regulatory B cell activation may promote T cell proliferation and differentiation of pro-inflammatory Th1 cells. Thus, both events lead to exacerbated inflammation in genetically-susceptibility KD patients. Allelic regulation of *BLK* expression in specific subtypes of regulatory B cells and the balance between effector and regulatory populations of both B cells and T cells requires further investigation at various disease stages of KD development.

We observed modest but significantly lower expression of *BLK* in the individuals carrying risk allele rs2736340. The sequence variations in or near the *BLK* gene have been shown to cosegregate with maturity-onset diabetes of the young (MODY) in familial studies. All variants identified from the MODY families were associated with a 60 to 80% decrease of *BLK* expression [20]. However, none of the family members carrying *BLK* mutations reported a history of autoimmune disorders. The specific mutations may have tissue-specific effects. On the other hand, the interaction between *BLK* with other genes and environmental conditions may be important in the susceptibility of KD and other autoimmune disorders.

In conclusion, we have validated the association between polymorphisms of the *BLK* locus and susceptibility to KD in Korean- and European-descended populations. Using a meta-analysis of Asian data, we have narrowed down the region most significantly associated with KD to the rs2736340-located LD block comprising the promoter region of *BLK*. Furthermore, we have provided evidence showing that the risk allele of rs2736340 is associated with lower expression of *BLK* in peripheral blood B cells during the acute stage of KD. Decreased BLK expression in peripheral blood B cells may alter B cell function and predispose individuals to KD. These associative data suggest a role for B cells during acute KD. Understanding the functional implications may facilitate the development of B cell-mediated therapy for KD.

## Supporting Information

File S1 **Contains:** Table S1. Association of genetic variants in the *BLK* region and Kawasaki disease in Han Chinese and Korean populations (two independent panels). Table S2. Association of genetic variants in the *BLK* region and Kawasaki disease in European descent GWAS. Table S3. Analysis the correlation of genotypes of tag SNPs in *BLK* region with *BLK* expression levels in transformed B cells in Han Chinese in Bejing. Figure S1. Linkage disequilibrium (LD) structure of region surrounding *BLK*. Relative position of genes mapping to the *BLK* region is based on NCBI Build 36. Pairwise LD plots of the estimated statistics of the square of the correlation coefficient (*r^2^*) are illustrated with Haploview software. The values in each diamond, which indicate the LD relationship between each pair of SNPs, were derived from genotypes in the Han Chinese GWAS. Red diamonds without a number represent *r^2^* = 1.
(PDF)
