# Supplementary material for: Replication and Meta-Analysis of GWAS Identified Susceptibility Loci in Kawasaki Disease Confirm the Importance of B Lymphoid Tyrosine Kinase (BLK) in Disease Susceptibility
Source: PLoS One. 2013 Aug 30;8(8):e72037. doi: 10.1371/journal.pone.0072037 (PMC3758326; doi:10.1371/journal.pone.0072037)
Supplement: File S1 — Contains: Table S1. Association of genetic variants in the BLK region and Kawasaki disease in Han Chinese and Korean populations (two independent panels). Table S2. Association of genetic variants in the BLK region and Kawasaki disease in European descent GWAS. Table S3. Analysis the correlation of genotypes of tag SNPs in BLK region with BLK expression levels in transformed B cells in Han Chinese in Bejing. Figure S1. Linkage disequilibrium (LD) structure of region surrounding BLK. Relative position of genes mapping to the BLK region is based on NCBI Build 36. Pairwise LD plots of the estimated statistics of the square of the correlation coefficient (r2) are illustrated with Haploview software. The values in each diamond, which indicate the LD relationship between each pair of SNPs, were derived from genotypes in the Han Chinese GWAS. Red diamonds without a number represent r2 = 1. (PDF) [file pone.0072037.s001.pdf]

**Supplementary information**

**Supplementary note**


**Taiwan Kawasaki Disease Genetics Consortium**

Jeng-Sheng Chang, Fuu-Jen Tsai (Department of Pediatrics, China Medical University Hospital, Taichung, Taiwan)
Ho-Chang Kuo, Chi-Di Liang, Kao-Pin Hwang (Department of Pediatrics, Kaohsiung Chang Gung Memorial Hospital, Kaohsiung, Taiwan)
Luan-Yin Chang, Li-Min Huang (Department of Pediatrics, National Taiwan University Hospital, Taipei, Taiwan)
Ming-Ren Chen, Hsin Chi, Fu-Yuan Huang, Nan-Chang Chiu (Department of Pediatrics, Mackay Memorial Hospital, Taipei, Taiwan)
Meng-Luen Lee (Department of Pediatrics and Divisions of Pediatric Cardiology, Changhua Christian Hospital, Changhua, Taiwan)
Yhu-Chering Huang (Division of Pediatric Infectious Diseases, Chang Gung Memorial Hospital at Linkou, Taoyuan, Taiwan)
Betau Hwang (Department of Pediatrics, Taipei City Hospital, ZhongXiao Branch, Taipei, Taiwan)
Pi-Chang Lee (Department of Pediatrics, Taipei Veterans General Hospital, Taipei, Taiwan)

**Korean Kawasaki Disease Genetics Consortium**

Jeong-Jin Yoo, In-Sook Park, Soo-Jong Hong, Kwi-Joo Kim (Department of Pediatrics, Asan Medical Center, Seoul, Korea)
Jong-Keuk Lee, Jae-Jung Kim, Young-Mi Park (Asan Institute for Life Sciences, University of Ulsan College of Medicine, Seoul, Korea)
Young Mi Hong, Saejung Sohn (Department of Pediatrics, Ewha Womans University Hospital, Seoul, Korea)
Gi Young Jang, Kee-Soo Ha, Hyo-Kyoung Nam, Jung-Hye Byeon (Department of Pediatrics, Korea University Hospital, Seoul, Korea)
Sin Weon Yun (Department of Pediatrics, Chung-Ang University Hospital, Seoul, Korea)
Myung Ki Han (Department of Pediatrics, University of Ulsan, Gangneung Asan Hospital, Gangneung, Korea)
Kyung-Yil Lee, Ja-Young Hwang, Jung-Woo Rhim (Department of Pediatrics, The Catholic University of Korea, Daejeon St. Mary's Hospital, Daejeon, Korea)
Min Seob Song (Department of Pediatrics, Inje University Paik Hospital, Busan, Korea)
Hyoung Doo Lee (Department of Pediatrics, Pusan National University Hospital, Busan, Korea)
Dong Soo Kim (Department of Pediatrics, Yonsei University College of Medicine, Severance Children's Hospital, Seoul, Korea)
Hong-Ryang Kil (Department of Pediatrics, Chungnam National University Hospital, Daejeon, Korea)
Gi-Beom Kim (Department of Pediatrics, Seoul National University Children's Hospital, Seoul, Korea)
Kyung Lim Yoon (Department of Pediatrics, Kyung Hee University Hospital at Gangdong, Seoul, Korea)
Jong-Duk Kim, Jae-Moo Lee (Seoul Clinical Laboratories, Seoul, Korea).

**International Kawasaki Disease Genetics Consortium**:
Australia: Miranda Odam, Frank Christiansen, University of Western Australia, Campbell Witt, Royal Perth Hospital, Perth, Western Australia; Paul Goldwater, The Women's & Children's Hospital, South Australia; Nigel Curtis, Royal Children's Hospital, Victoria; Pamela Palasanthiran, John Ziegler, Sydney Children's Hospital, Randwick, New South Wales; Michael Nissan, Royal Children's Hospital, Clare Nourse,
Mater Hospital, Queensland
The Netherlands: Irene M Kuipers, Jaap J Ottenkamp, Judy Geissler, Maarten Biezeveld,
Emma Children's Hospital, Academic Medical Center; Luc Filippini, Juliana Children's
Hospital, The Hague
Singapore: Ling Ling
United Kingdom: Michael Levin, Victoria J. Wright, Imperial College, London Paul Brogan, Nigel Klein, Vanita Shah, Michael Dillon, The Institute of Child Health, London; Robert Booy, Delane Shingadia, Anu Bose, Thomas Mukasa,
Royal London Hospital, London; Robert Tulloh, Bristol Royal Hospital for Children, Bristol; Colin Michie, Ealing Hospital, London.

## Supplementary Tables and Figures

Table S1. Association of genetic variants in the *BLK* region and Kawasaki disease in Han Chinese and Korean populations (two independent panels).

| | | | | | | Taiwan GWAS | | | | Korean GWAS | | | | Joint |
|---|---|---|---|---|---|---|---|---|---|---|---|---|---|---|
| **SNP** | **Chr** | **Position** | **Gene** | **Allele** | **Risk Allele** | **RAF Control (*n* = 1107)** | **RAF KD Cases (*n* = 622)** | **OR (95%CI)** | ***P* values** | **RAF Control (*n* = 600)** | **RAF KD Case (*n* = 186)** | **OR (95% CI)** | ***P-values*** | **TW_KR** |
| rs10109491 | 8 | 1349676 | BLK | AT | A | 0.623 | 0.690 | 1.350 (1.164–1.565) | 6.88E-05 | 0.554 | 0.562 | 1.031 (0.815–1.303) | 7.95E-01 | 5.92E-04 |
| rs2736340 | 8 | 11381382 | BLK | CT | T | 0.722 | 0.797 | 1.514 (1.282–1.789) | 8.74E-07 | 0.694 | 0.766 | 1.446 (1.104–1.895) | 5.23E-03 | 9.25E-08 |
| rs1478900 | 8 | 11385069 | BLK | CT | T | 0.719 | 0.785 | 1.423 (1.208–1.676) | 2.52E-05 | 0.713 | 0.777 | 1.399 (1.064–1.841) | 1.28E-02 | 5.15E-06 |
| rs2618476 | 8 | 11389950 | BLK | AG | G | 0.725 | 0.797 | 1.491 (1.262–1.762) | 2.23E-06 | 0.700 | 0.761 | 1.363 (1.042–1.782) | 1.92E-02 | 7.70E-07 |
| rs998683 | 8 | 11390409 | BLK | AG | A | 0.712 | 0.781 | 1.448 (1.230–1.705) | 8.20E-06 | 0.690 | 0.750 | 1.348 (1.032–1.760) | 2.40E-02 | 3.24E-06 |
| rs998682 | 8 | 11390461 | BLK | AG | G | 0.738 | 0.804 | 1.458 (1.231–1.727) | 1.06E-05 | 0.727 | 0.783 | 1.357 (1.026–1.795) | 2.59E-02 | 4.41E-06 |
| rs1478895 | 8 | 11390744 | BLK | CG | G | 0.738 | 0.805 | 1.459 (1.232–1.728) | 1.09E-05 | 0.726 | 0.788 | 1.401 (1.060–1.852) | 1.40E-02 | 2.54E-06 |
| rs2618479 | 8 | 11393230 | BLK | CT | C | 0.72 | 0.789 | 1.453 (1.232–1.713) | 1.01E-05 | 0.726 | 0.788 | 1.402 (1.061–1.854) | 1.35E-02 | 2.30E-06 |
| rs2736354 | 8 | 11406140 | BLK | AG | G | 0.724 | 0.785 | 1.392 (1.181–1.640) | 8.19E-05 | 0.710 | 0.788 | 1.522 (1.151–2.013) | 2.89E-03 | 3.84E-06 |
| rs6993775 | 8 | 11407398 | BLK | AC | A | 0.719 | 0.783 | 1.409 (1.197–1.660) | 3.86E-05 | 0.706 | 0.785 | 1.521 (1.153–2.006) | 2.62E-03 | 1.73E-06 |
| rs1382566 | 8 | 11422250 | BLK | CG | G | 0.738 | 0.800 | 1.418 (1.198–1.679) | 4.40E-05 | 0.731 | 0.819 | 1.663 (1.240–2.232) | 6.08E-04 | 4.93E-07 |
| rs17153419 | 8 | 11431642 | BLK | CT | C | 0.715 | 0.78 | 1.409 (1.197–1.659) | 3.52E-05 | 0.714 | 0.785 | 1.461 (1.107–1.928) | 7.17E-03 | 4.09E-06 |
| rs1478897 | 8 | 11432641 | BLK | AT | T | 0.722 | 0.784 | 1.397 (1.186–1.646) | 5.60E-05 | 0.735 | 0.804 | 1.477 (1.109–1.966) | 7.42E-03 | 6.52E-06 |

Case RAF, risk allele frequency in Kawasaki disease cases; Control RAF, risk allele frequency in controls; TW_KR, meta-analysis of Taiwan and Korean GWAS.

Table S2. Association of genetic variants in the *BLK* region and Kawasaki disease in European descent GWAS.

| SNP | Chr | Position | Risk Allele | RAF Case | RAF Control | OR (95%CI) | *P* value | |
|---|---|---|---|---|---|---|---|---|
| rs2898280 | 8 | 11358702 | G | 0.546 | 0.496 | 1.21 (1.05–1.40) | 0.011 | genotyped |
| rs6989817 | 8 | 11367774 | A | 0.399 | 0.447 | 0.84 (0.72–0.98) | 0.023 | genotyped |
| rs12680762 | 8 | 11369436 | A | 0.330 | 0.275 | 1.25 (1.07–1.47) | 0.0063 | genotyped |
| rs2736340 | 8 | 11381382 | A | 0.290 | 0.251 | 1.19 (1.01–1.41) | 0.037 | genotyped |
| rs13277113 | 8 | 11386595 | A | 0.286 | 0.247 | 1.19 (1.01–1.40) | 0.043 | genotyped |
| rs2618476 | 8 | 11389950 | C | 0.284 | 0.246 | 1.18 (1.00–1.39) | 0.056 | imputed |

All point estimates (per-allele OR and its corresponding *P* value) have been adjusted for the top four axes of genetic stratification, as previously described[6].
Case RAF, risk allele frequency in Kawasaki disease cases; Control RAF, risk allele frequency in controls.

Table S3. Analysis the correlation of genotypes of tag SNPs in *BLK* region with *BLK* expression levels in transformed B cells in Han Chinese in Bejing.

| SNP | | | | | P value |
|---|---|---|---|---|---|
| rs2736340 | Geno | C/C | C/T | T/T | |
| | Counts | 3 | 17 | 23 | $1.379\times10^{-4}$ |
| | Freq | 0.070 | 0.395 | 0.535 | |
| | Mean | 10.630 | 10.350 | 9.647 | |
| | SD | 0.583 | 0.470 | 0.644 | |
| rs6993775 | Geno | G/G | G/T | T/T | |
| | Counts | 2 | 16 | 25 | $8.27\times10^{-3}$ |
| | Freq | 0.047 | 0.372 | 0.581 | |
| | Mean | 10.790 | 10.310 | 9.725 | |
| | SD | 0.730 | 0.437 | 0.689 | |
| rs1382566 | Geno | C/C | C/G | G/G | |
| | Counts | 2 | 17 | 24 | $6.342\times10^{-3}$ |
| | Freq | 0.465 | 0.395 | 0.558 | |
| | Mean | 10.070 | 10.380 | 9.711 | |
| | SD | 0.286 | 0.485 | 0.695 | |

Geno, genotypes; Freq, frequency; SD, standard deviation.

A

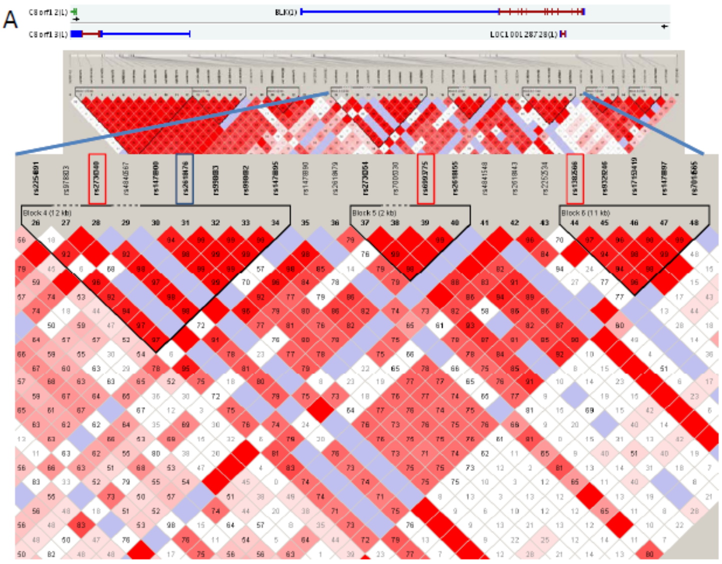

B

| rs2736340 | D' | R2 |
| --- | --- | --- |
| rs6993775 | 0.803 | 0.621 |
| rs1382566 | 0.766 | 0.562 |

Figure S1. Linkage disequilibrium (LD) structure of region surrounding *BLK*. Relative position of genes mapping to the *BLK* region is based on NCBI Build 36. Pairwise LD plots of the estimated statistics of the square of the correlation coefficient ($r^2$) are illustrated with Haploview software. The values in each diamond, which indicate the LD relationship between each pair of SNPs, were derived from genotypes in the Han Chinese GWAS. Red diamonds without a number represent $r^2$ = 1.
